# Supplementary material for: The relationship between participation in leisure activities and incidence of falls in residential aged care
Source: PLoS One. 2024 Apr 25;19(4):e0302678. doi: 10.1371/journal.pone.0302678 (PMC11045105; doi:10.1371/journal.pone.0302678)
Supplement: Attachment — Submitted filename: Response to Reviewers.docx [file pone.0302678.s001.docx]

To the Editor:

We appreciate the opportunity to address the Editors’ and Reviewers’ comments and revise our manuscript. Below, please find item-by-item responses to the Editors’/Reviewers’ comments, which are included verbatim. All page and paragraph numbers refer to locations in the revised manuscript.

**EDITOR COMMENTS/JOURNAL REQUIREMENTS**

**COMMENT 1**:

When submitting your revision, we need you to address these additional requirements.

Please ensure that your manuscript meets PLOS ONE's style requirements, including those for file naming. The PLOS ONE style templates can be found at

<https://journals.plos.org/plosone/s/file?id=wjVg/PLOSOne_formatting_sample_main_body.pdf> and

<https://journals.plos.org/plosone/s/file?id=ba62/PLOSOne_formatting_sample_title_authors_affiliations.pdf>

**RESPONSE 1:**

Thank you for this comment. The manuscript has now been revised carefully according to PLOS ONE's style requirements. All revisions have been highlighted in the revised manuscript (change-tracked version).

**COMMENT 2**:

Thank you for stating the following financial disclosure:

"This work was supported by the Australian National Health and Medical Research Council (NHMRC) Partnership Project Grant (1170898). The funding organization did not have any influence on the study design, data collection, analysis and interpretation as well as the preparation, review, or approval of the manuscript for publication."

Please state what role the funders took in the study. If the funders had no role, please state: "The funders had no role in study design, data collection and analysis, decision to publish, or preparation of the manuscript."

If this statement is not correct you must amend it as needed.

Please include this amended Role of Funder statement in your cover letter; we will change the online submission form on your behalf.

**RESPONSE 2:**

Thank you for the comment. In the financial disclosure of the online system, we have now specified that ‘The funders had no role in study design, data collection and analysis, decision to publish, or preparation of the manuscript’.

**COMMENT 3:**

In the online submission form, you indicated that [The data used in this study are available from the aged care provider (Anglicare), but restrictions apply to the availability of these data, which were used under license for the current study, and so are not publicly available. Data are however available from the authors (Guogui Huang, guogui.huang@mq.edu.au) upon reasonable request and with permission of Anglicare.].

All PLOS journals now require all data underlying the findings described in their manuscript to be freely available to other researchers, either 1. In a public repository, 2. Within the manuscript itself, or 3. Uploaded as supplementary information.

This policy applies to all data except where public deposition would breach compliance with the protocol approved by your research ethics board. If your data cannot be made publicly available for ethical or legal reasons (e.g., public availability would compromise patient privacy), please explain your reasons on resubmission and your exemption request will be escalated for approval.

**RESPONSE 3:**

Thank you for this comment. The data used in this paper is managed by the Data Management Plan approved by Macquarie University Human Research Ethics Committee Medical Sciences Committee (Ref No: 52019614412614). According to the Plan, ‘All research data (e.g. interviews, data extractions) will be stored electronically on Macquarie University’s secure password-protected server’, and ‘only the named project team members approved on the ethics application will have access to the data extracted and collected during this project’. Therefore, given the ethical restrictions, we are unable to make the data publicly available through public deposition.

To increase clarity, we have now provided more information about data availability and data restrictions in the revised manuscript.

‘The data used in this study were collected by the aged care provider and restrictions apply to the availability of these data. As part of the approval issued by Macquarie University Human Research Ethics Committee Medical Sciences Committee (Ref No: 52019614412614), ‘All research data (e.g. interviews, data extractions) will be stored electronically on Macquarie University’s secure password-protected server’ and ‘Only the named project team members approved on the ethics application will have access to the data extracted and collected during the period of the research project’, given that data contain potentially identifying or sensitive patient information. Therefore, the data used in this study cannot be deposited in a public data repository. Data are however available from the authors (Guogui Huang, guogui.huang@mq.edu.au) or through the Centre of Health Systems and Safety Research, Macquarie University ([chssr@mq.edu.au](mailto:chssr@mq.edu.au)) upon reasonable request and with permission of aged care provider.’ (page 2 lines 29-41)

**COMMENT 4:**

Please include your full ethics statement in the ‘Methods’ section of your manuscript file. In your statement, please include the full name of the IRB or ethics committee who approved or waived your study, as well as whether or not you obtained informed written or verbal consent. If consent was waived for your study, please include this information in your statement as well.

**RESPONSE 4:**

Thank you for this comment. We have now included the following statements to provide IRB information and extra ethics information in the section of method of the revised manuscript.

“The study was reviewed and approved by Macquarie University Human Research Ethics Committee (Application no. 52019614412614).” (page 8 lines 166-168)

“Because de-identified data were used, requirement of obtaining participants’ consent was waived by Macquarie University Human Research Ethics Committee.” (page 8 lines 171-173)

**COMMENT 5:**

Please ensure that you refer to Figure 2 in your text as, if accepted, production will need this reference to link the reader to the figure.

**RESPONSE 5:**

Thank you for this comment. The content of Figure 2 was originally presented as Table 4 in the early version of the paper. However, we decided to transfer information in Table 4 to Figure 2 in the final submitted version for better visualisation purposes. Therefore, all the references of Table 4 in the original manuscript actually refer to Figure 2. We have now corrected this error in the revised manuscript.

**COMMENT 6:**

Please include a copy of Table 4 which you refer to in your text on page 13.

**RESPONSE 6:**

Thank you for this reminder. Please refer to our response to the Comment 5.

**Reviewer #1:**

**COMMENT 1:**

Thank you for the opportunity to review this manuscript, titled ‘The relationship between participation in leisure activities and fall incidence in residential aged care’. The study found an association between quantity of leisure activities (exercise and non-exercise), and reduced fall risk. The study found this association was stronger in residents living with dementia, compared to those not living with dementia. The manuscript was well written and framed. I understand my comments below may be limited by data availability.

Methods: have the falls outcomes in this dataset been validated at all?

**RESPONSE 1:**

Thank you for this comment. We used routinely collected falls incident data by the aged care provider. Staff of residential aged care facilities recorded falls incidents via an incident registry form and documented the details of falls, including categorical variables (e.g., facility, type of incident, location, hospitalisation, and severity assessment code score), continuous variables (e.g., time and date), and open text fields (e.g., incident details and outcomes of incident review). The fall outcomes were validated by registered nurses or aged care staff and were reported to facility manager afterwards.

The aged care provider has contributed substantial financial, staffing and facility/device resources to collect falls incident data. Falls incidents must be reported as part of the National Aged Care Mandatory Quality Indicator Program every three months in Australia. A recent study (<https://pubmed.ncbi.nlm.nih.gov/38217881/>) has validated and demonstrated substantial agreement between incident data (e.g., falls incident dataset) and progress records in residential aged care setting of Australia.

**COMMENT 2:**

I note that the leisure activities data contains “residents’ participation level” - can this be used to say anything about dose-response? Firstly, for example, activities that have some sort of meaning to the participant vs something to pass the time. Were there enough ‘attended but refused to participate’ to investigate around this? Why would residents attend but not participate - do residents self-select activities? Who drives leisure activities? Do RACFs have targets or KPIs around activities engagement? Line 381 – raises also the question of ‘quality’ of activity or level of participation

Secondly, do you have data to investigate whether there was a difference in time spent on leisure activities? E.g. between 15 mins vs 5 hours of non-exercise leisure?

Thirdly, can you look into variety at all? E.g. going to Church 3x/week vs doing 3 different activities/week? E.g. one strong social network vs a potentially wider, more varied one?

**RESPONSE 2:**

Thank you for this insightful comment. We initially considered using the variable of participation level when conducting the analysis. However, participation level was missing for 5.6% of the participants. For the rest 94.4% that had participation level, 97.6% was recorded as ‘Participated’, 1.6% as ‘Participation with encouragement’, 0.7% as ‘Refused participation’ and 0.1% as ‘Unusual behaviour’. With a disproportionally high presence of ‘Participated’, the variable of participation level cannot be used to sufficiently differentiate the level of leisure participation among residents and hence was not used in the final analysis.

Regarding the length of time spent in each leisure participation, unfortunately, such information is missing in the dataset because only the starting time of leisure participation was recorded in the care providers’ dataset while the ending time was not documented. We have now recognised this as a research limitation in the discussion section.

The correspondingly revised text in the revised manuscript is presented below.

‘Fourth, given that the aged care provider did not record the ending time of each leisure participation, we are unable to incorporate the length of time spent in each leisure activity in our analysis; this might miss the differences in the extent/level of participation among different residents.’ (page 20 lines 406-409)

Finally, we initially intended to examine how the relationship between leisure participation and falls vary by type of leisure activities. However, it is difficult to group certain common leisure activities into a specific category. For example, for some common leisure activities, such as movie/music/book club activities, we are unable to define them into leisure activities for social purposes or leisure activities for entertainment purposes. Therefore, we adopt the division of ‘exercise’ and ‘non-exercise’, but have not further divided ‘non-exercise’ into more detailed categories.

**COMMENT 3:**

Classification – ‘medium high’ vs ‘high’ – doesn’t take into account time spend on leisure activity, i.e. 1 activity for 5 hrs vs 3 activities for 20 mins each? More time taken in activities = more time around others who can help watch them/prevent falls during that time (i.e. less time at risk)

It might also be helpful to put these classifications in terms that are easier to grasp e.g. Low is no activities to an activity every 4.42 days (this leads to the question: would it be better to have classifications that make more practical sense? You could end up with uneven sized groups but x activities every (whole) day is easier to understand and interpret?

**RESPONSE 3:**

Thank you for this comment. As included in our response to the previous comment, we are unable to measure the length of time spent in each leisure participation as this data is not available in the dataset. This has now been recognised as a research limitation in the revised manuscript.

‘Fourth, given that the aged care provider did not record the ending time of each leisure participation, we are unable to incorporate the length of time spent in each leisure activity in our analysis; this might miss the differences in the extent/level of participation among different residents’ (page 20 lines 406-409)

We appreciate the reviewer’s suggestion of using more easy-understand expressions for the classification of leisure participation. This suggestion has now been adopted in the revised manuscript. Specifically, we transferred the original expression of falls rate per 1,000 resident days into falls rate per week, which is more easily understood. Additionally, we also presented the frequent of leisure participation in every certain number of days (e.g., no activities to an activity every 4.43 days), following reviewer’s suggestion. Moreover, we have now specified the number of leisure activities in every certain number of days following most appearances of ‘low’, ‘medium’, ‘medium high’ and ‘high’ in the text. This will facilitate readers better interpret the corresponding meaning.

Some of the revised text is presented below.

‘We used the three quartile values of the leisure participation rate to classify leisure participation level into four categories: low (from the lowest level of leisure participation rate to the first quartile, i.e., 0-1.59 activities per week, which equates to a range of no activities to an activity every 4.43 days, for all sample); middle (from the first quartile to the second quartile, i.e., 1.59-3.01 activities per week, or an activity every 2.33-4.43 days, for all sample); middle-high (from the second quartile to the third quartile, i.e., 3.01-5.32 activities per week, or an activity every 1.32-2.33 days, for all sample) and high (from the third quartile to the highest level of leisure participation rate, i.e., 5.32-14.31 activities per week, or an activity in less than 1.32 days, for all sample).’ (page 11 lines 221-229)

‘For example, compared to those with a low level of leisure participation (i.e., <25th percentile, or no activities to an activity every 4.43 days), residents in high leisure participation group (i.e., ≥75th percentile, or an activity in less than 2.33 days) were 26% less likely to experience all falls (IRR at 0.74 [95% CI=0.60, 0.91]) and were 40% less likely to experience injurious falls (IRR at 0.60 [95% CI=0.47, 0.76]).’ (page 15 lines 274-278)

**COMMENT 4:**

There's some mention of loneliness in your ms, but not much? This emerging field/concept seems quite relevant (and related to my comment #2 in terms of quality and time of activities).

**RESPONSE 4:**

Thank you for this comment. As pointed out by the reviewer, loneliness is an important mental health condition among older people living in RACF settings. A high prevalence rate of loneliness in RACFs has been reported previously, mainly due to factors such as the move to an unfamiliar environment, loss of previous connections and networks and deterioration of physical health.

In our study, the mental health status of a RACF resident is measured by the level of depression and anxiety, two mental health indicators which are closely related to the level of loneliness. Although loneliness is not directly gauged and recorded in the aged care provider’s dataset, the level of depression and anxiety were both controlled for in our multilevel negative binomial regression, and might co-reflect the loneliness status of a resident.

Since the role of loneliness in the relationship between leisure participation and falls is not the focus of the present paper, we have not discussed extensively about the loneliness status and how it might affect falls and quality of life. However, it would be of interest for us to investigate in the next step of our investigation.

**COMMENT 5:**

Please clarify numbers in Fig 1: leisure activity data for n=3747, the missing n=35 didn’t do any activities or were they missing data? Same for falls data n=2519, missing n=1263. Also N=762 excluded (for interim care, discharged on same date etc), n=3782-762=3020 which is > final sample 3024?

**RESPONSE 5:**

Thank you for pointing out this confusion. The dataset of leisure activities only includes residents who ever participated in leisure activities during their admissions (n=3,747). Therefore, for those 25 residents in the dataset without any leisure participation recorded, they were treated as 0 leisure activities per 1000 resident days in the analysis. Similarly, the fall incident dataset only documents information of residents having experienced fall events (n=2,519); therefore, the remaining 1,263 residents in the profile dataset without fall incident records, they were treated as non-fallers in the analysis (i.e., 0 falls per 1000 resident days).

Moreover, for the mismatch of 3782-762=3020 vs 3024, there are two reasons: 1) 762 is the number of admissions rather than the number of residents; and 2) a few residents had multiple admissions over the study period. So for those with multiple admission records, even one single record of their admissions was removed, they were still be included in the analysis due to the rest of their admission records.

We have now provided a note under Fig 1 to present such explanations to increase clarity.

The newly added note is presented as below.

‘Note: 1, Dataset of leisure activities does not include information of residents without any attendance of leisure activities; 2, Dataset of fall incidents does not include information of residents who did not experienced any fall events; 3, Some residents had multiple admissions during the study period, therefore, the number of residents in final sample was 3024, rather than 3020 (=3782-762).’

**COMMENT 6:**

Table 4 (mentioned in line 282) doesn’t exist? I think this refers to Fig 2?

**RESPONSE 6:**

Thank you for identifying this error. Table 4 actually refers to Fig 2. As our response to Comment 5 of editor explained, during our final revision before submission to PLOS ONE, we decided to convert the information in Table 4 to Fig 2 for better visualisation effect; however, we did not update the text correspondingly. We have now corrected this error in the revised manuscript.

**COMMENT 7:**

Would be good to see the results refered to in Fig 2 note 2 - perhaps in supp material?

**RESPONSE 7:**

Thank you for identifying this error. Please refer to our response to the previous comment.

**Reviewer #2:**

**COMMENT 1:**

I appreciate the effort put into your manuscript on the association between leisure activities and fall incidence. After careful consideration, I find that the paper requires a major revision for the following reasons:

Clarity in Methodological Justification:

In the Introduction section, the manuscript emphasizes the significance of considering the impacts of both dementia and "depression" on the association between leisure activities and fall incidence. However, the rationale for performing statistical analyses specifically on the basis of dementia is vaguely explained. It is crucial to either include the analysis related to depression or provide a more detailed and explicit logic in the Introduction section, focusing on dementia. This will enhance the overall coherence and comprehensibility of the study design.

**RESPONSE 1:**

Thank you for this constructive comment. To avoid confusion and improve the overall coherence of the study, we have now removed the content of depression in the introduction in the revised manuscript. Instead, we focus on the discussion on dementia and how this condition might critically affect an association between leisure participation and falls. We have cited more relevant literature to strengthen our discussion.

The revised text is presented below.

‘The overall positive effect of non-exercise leisure participation on individuals’ physical and psychological outcomes, well documented in the existing literature [11,27-30], might subsequently positively affect fall outcomes. Specifically, engagement in both exercise and non-exercise leisure activities (e.g., reading, playing board games and playing musical instruments) were found to be significantly associated with a reduced risk of dementia [30], which might therefore affect susceptibility to falls. For example, a recent systematic review that covers 38 longitudinal studies, with 2,154,818 participants at baseline, indicated that physical exercises (e.g., walking and jogging) (rate ratio=0.83, 95% CI 0.78–0.88), cognitive activities (e.g., reading, playing games and playing music instruments) (rate ratio=0.77; 95% CI 0.68–0.87) and social activities (e.g., participating in volunteer work and meeting relatives or friends) (rate ratio=0.93; 95% CI 0.87–0.99) were all associated with a decreased incidence of all-cause dementia [31]. Dementia is a strong predictor of falls due to a greater risk of people with dementia experiencing problems with mobility and balance and difficulties with their memory, along with side effect of medication prescribed for dementia (e.g., dizziness and sedation), behavioural and psychological symptoms associated with dementia (e.g., agitation) and challenges for individuals with dementia to navigate complex or unfamiliar environments [32,33]. The inverse relationship between participation in exercise and non-exercise leisure activities and dementia implies a potential positive impact of these activities on falls.’ (pages 6-7 lines 123-142)

**COMMENT 2:**

Lack of Data and Limitation Acknowledgment:

The manuscript categorizes leisure activities into "exercise activity" and "non-exercise activity" without presenting data on participants' physical function. This classification might be dependent on physical function, and the relevant data should be shown and included in the analyses in the study.

**RESPONSE 2:**

Thank you for this comment. As the reviewer points out, residents’ physical function could affect the type of leisure activities that residents intend to participate in. However, since a resident might participate in both exercise and non-exercise activities (n=3,022 for participants of non-exercise activities and n=2,504 for participants of exercise activities in our study), the group of participants of exercise activities and the group of participants of non-exercise participants are largely overlapped in the sample. Additionally, leisure participation could occur at any time points of admission, so we will need longitudinal data of physical functional status at each time of leisure participation to control the status of physical function. Given that we only have data of residents’ health function at admission, we are unable to achieve this. We have now recognised this as a research limitation in the discussion.

The revised text is presented below.

‘Seventh, residents’ decision to engage in exercise or non-exercise activities might be affected by their physical functional status, which could vary over the period of admission. Given a lack of longitudinal data, we were unable to control for any differences in the physical functional status in relation to engagement in exercise and non-exercise activities in our analysis.’ (pages 20-21 lines 416-420)
